# Analysis of Educational Mental Health and Emotion Based on Deep Learning and Computational Intelligence Optimization

**DOI:** 10.3389/fpsyg.2022.898609

**Published:** 2022-06-17

**Authors:** Junli Liu, Haoyuan Wang

**Affiliations:** ^1^School of Marxism, Xi’an Technological University, Xi’an, China; ^2^Faculty of Science, McMaster University, Hamilton, ON, Canada

**Keywords:** deep learning, computational intelligent optimization, educational mental health, emotional analysis, psychological

## Abstract

Understanding students’ psychological pressure and bad emotional reaction can solve psychological problems as soon as possible and avoid affecting students’ normal study life. With the improvement of global scientific and technological strength, and the step-by-step in-depth research on deep learning and computational intelligence optimization. Now, we have enough conditions to build a psychological and emotional data set for the field of education, and build a mental health stress detection model with emotional analysis function. In addition, a variety of experimental methods are used for comparison, which shows the superior performance of the model in practical application scenarios. The results show that: (1) the data set constructed for the model is reasonable. Psychological stress test shows that the tested college students are in good health and have no positive performance. Schools need to pay special attention to obsessive–compulsive disorder and interpersonal sensitivity, and the average values of both indicators are higher than 0.9. (2) For the optimization of ant colony algorithm (ACO) computational intelligence, both the stability and the average execution time of the algorithm are obviously higher than those of other algorithms. This model has obvious performance advantages after using this algorithm. (3) Using loss function value to measure the difference between simulated emotion analysis and real value. The difference of most emotion tests is less than 3%; the accuracy difference between sadness and fear is about 7%. Although the final results prove the feasibility of this method, there are still some shortcomings to be optimized.

## Introduction

Psychological side effects accompanying emotions will also make students physically uncomfortable, resulting in emotional instability, maladjustment, tension, and anxiety. So that students can make negative behaviors with adverse effects in behavior and speech. Reasonable and effective use of the scientific and technological power in the computer field, so that machines or models can identify human psychological state and emotions. This is a hot research direction. Although human beings can hide their emotions to a certain extent, they cannot control their body signals. Using this feature, this paper can identify students’ real emotional state more accurately and accurately, so as to obtain more comprehensive and targeted mental health research.

For colleges and universities, the lack of teachers and heavy teaching tasks make it difficult to take care of each student’s emotional problems. However, the fierce competitive life and the cruelty of society make students’ pressure increasing day by day. In addition, due to individual differences, each student’s performance is different, so it is impossible to accurately perceive students’ psychological stress experience only by manpower. With the latest development of deep learning, people have made many achievements. In this paper, multi-channel human physiological signals will be used for feature fusion detection and analysis. The following literature can give some theoretical support and data support to this paper.

The research in this paper has certain theoretical and practical significance. Collecting literature, we can find that there are few researches on the construction of college students’ psychology in China. However, there are fewer models or methods to analyze students’ emotions and explore the root causes. This paper refers to some materials related to the research content of this paper. Specific contents are as follows: When judging emotional tendentiousness, emotional words and emoticons are considered, and a text emotion analysis model is proposed (Jia et al., 2013). A Chinese text emotion classification model for user comments is constructed by word embedding and cyclic neural network (Diao et al., 2021). An emotion classification model based on convolution neural network and cyclic neural network is proposed (Song and Yan, 2020). From the perspective of multiple intelligences theory, this paper explores the mode of mental health education in colleges and universities (Zhang, 2021). Using the attribute reduction algorithm based on information entropy in rough set theory, this paper studies the mental health of college students (Xu et al., 2020). Based on cloud computing technology, an online service platform for college mental health is designed to evaluate college students’ emotions (Li, 2021). Combing the ideological and political materials of the course, comprehensively evaluating the optimization of online teaching evaluation index of mental health education course (Qin, 2021). Based on deep learning, an efficient lightweight U-Net semantic segmentation model is constructed to quantitatively analyze the fear emotional behavior of mice (Liu et al., 2021). Compare various deep network architectures, and apply deep learning technology to the study of emotional EEG data (Li et al., 2020). Effectively fuse the emotional information expressed by facial expressions and body postures for emotional recognition (Wen et al., 2021). BERT model based on deep learning is classified into three categories to analyze netizens’ comments on Weibo during COVID-19 epidemic (Liu, 2020). A student public opinion analysis system based on long-term and short-term memory (LSTM)-CNN hybrid model is proposed and implemented (Huang and Sun, 2020). This paper proposes an online analysis method of students’ emotions based on artificial intelligence technology to help understand students’ learning status (Tang et al., 2021). Prediction of mental health problems of higher vocational students through data preprocessing, BP algorithm, and deep learning technology (Yang and Xu, 2020). The mechanism model of deep learning is preliminarily constructed, and the learning status index is set to achieve accurate evaluation (Ma et al., 2022). Some achievements have been made in the analysis of educational mental health and emotion by some simple technologies in above literatures. The application of platform or the statistical method of students’ online learning in the above methods can solve the existing problems, but cannot objectively analyze the theoretical characteristics and emotional expression of educators. In the deep learning method proposed in this paper, intelligent computing model is used to optimize the related learning parameters, which can quickly obtain the optimal feature effect, and has good prediction accuracy and efficiency.

## Theoretical Basis

### Deep Learning

Its full name is Deep Learning (Hu et al., 2022). Since deep learning was introduced into machine learning, it belongs to a new research direction closely related to artificial intelligence. Its learning algorithm is complex and its models are diverse and rich. Up to now, great achievements have been made in image and speech recognition, and they are widely used in medical treatment, transportation, education, and other fields. It has greatly improved people’s work, study, and life, and made people enjoy more efficient and convenient new technologies. In recent years, deep learning has achieved great success, which has solved many complex pattern recognition problems accumulated by predecessors and promoted a big step forward in the field of artificial intelligence.

There are two training processes for deep learning. One is unsupervised learning from the bottom layer to the top layer. The other is to use tagged data. Train the data from the top down. While transmitting errors, fine-tuning the network is carried out.

Matrix expression neuron:


(1) $$h_{\theta }\left(\theta^{T}x\right)=f\left(\theta_{0}*1+\theta_{1}x_{1}+\theta_{2}x_{2}+\ldots \right)=f\left(\theta^{T}x\right)$$

In addition, for the excitation function. There are several common types:

1. Sigmoida Function
(2) $$f\left(x\right)=\frac{1}{1+e^{-x}}$$
2. ReLu Function (Ren, 2021)
(3) ReLu(x)=max(0,x)
3. Tanh Function (Jiang, 2020)
(4) tanh(x)=2sigmoid(2x)−1

In order to better explain the feedforward process of neural network.


(5) $$a_{1}^{\left(2\right)}=f\left(\theta_{10}^{\left(1\right)}x_{0}+\theta_{11}^{\left(1\right)}x_{1}+\theta_{12}^{\left(1\right)}x_{2}+\theta_{13}^{\left(1\right)}x_{3}\right)$$

(6) $$a_{2}^{\left(2\right)}=f\left(\theta_{20}^{\left(1\right)}x_{0}+\theta_{21}^{\left(1\right)}x_{1}+\theta_{22}^{\left(1\right)}x_{2}+\theta_{23}^{\left(1\right)}x_{3}\right)$$

(7) $$a_{3}^{\left(2\right)}=f\left(\theta_{30}^{\left(1\right)}x_{0}+\theta_{31}^{\left(1\right)}x_{1}+\theta_{32}^{\left(1\right)}x_{2}+\theta_{33}^{\left(1\right)}x_{3}\right)$$

(8) $$h_{\theta }\left(x\right)=a_{1}^{\left(3\right)}=f\left(\theta_{10}^{\left(2\right)}a_{0}{}^{\left(2\right)}+\theta_{11}^{\left(2\right)}a_{1}{}^{\left(2\right)}+\theta_{12}^{\left(2\right)}a_{2}{}^{\left(2\right)}+\theta_{13}^{\left(2\right)}a_{3}{}^{\left(2\right)}\right)$$

where $a_{i}^{\left(j\right)}$ represents the result of the activation function; It means that the *i*-th neuron in layer *j* receives the input from layer *j*-1. $\theta_{mn}^{\left(j\right)}$ represents the connection weight of the nth neuron; from the *m* neuron in layer *j* to the *j* + 1 layer. In addition, this article treats bias as a parameter, and corresponds to the connection input 1.

### Computational Intelligence Optimization

Computational intelligence is also closely related to deep learning and artificial intelligence. With the help of computer technology and related tools, computational intelligence can simulate human intelligence, so as to obtain and utilize various data information. Its function is very applicable to the study of people’s psychology and emotion. In addition, for many algorithms in the computational intelligence system, the mechanism can be internally optimized. This paper takes the optimization of ant colony algorithm (ACO) as an example, and introduces this algorithm to analyze the data processing. Computational intelligence is the deepening and development of artificial intelligence. This is a very promising hot research topic. It can use neural network model to open a new research approach, simulate psychological process, and restore human psychological activity state. Improve the operation efficiency of the algorithm in practical application. As shown below:

1. Initialize ant colony algorithm. Set the parameters.
2. Choose city and transfer probability.
(9) $$p_{ij\left(k\right)}=\{\begin{array}{l} \frac{\tau_{ij}^{\alpha }\left(t\right)\eta_{ij}^{\beta }}{\sum \tau_{is}^{\alpha }\left(t\right)\eta_{is}^{\beta }}\\ 0 \end{array}j,s\in tabu_{\left(k\right)}$$
3. Principles for updating global information.
(10) $$\tau_{ij}\left(t+n\right)=\rho \cdot \tau_{ij}\left(t\right)+\Delta \tau_{ij}$$
(11) $$\Delta \tau_{ij}=\overset{m}{\underset{k=1}{\sum }}\Delta \tau_{ij}\left(k\right)$$
(12) $$\Delta \tau_{ij}\left(k\right)=\{\begin{array}{l} \frac{Q}{L_{k}}\\ 0 \end{array}$$

Convergence analysis:

1. Enough iterations:
(13) $$\underset{t\to \infty }{\lim}\tau_{ij}^{*}\left(t\right)=\bar{\tau }=\frac{Q\cdot m}{\left(1-\rho \right)\cdot X_{\min}}$$
(14) $$\underset{t\to \infty }{\lim}\tau_{ij}\left(t\right)\leq \bar{\tau }$$
2. Finite number of iterations:
(15) $$\tau_{ij}\left(t\right)\geq \underline{\tau }=\rho_{\max}^{g}\cdot \tau_{ij}\left(0\right)$$
(16) $$\tau_{ij}\in \left[\underline{\tau },\bar{\tau }\right]$$

### Signal Processing Technology

#### Time Domain Analysis

From a statistical point of view, the original time series signal is analyzed. For the calculation of sequence data, as follows:

Average (Qiu et al., 2020):


(17) $$mean=\frac{1}{N}\overset{N}{\underset{n=1}{\sum }}x_{n}$$

Standard deviation (Wang et al., 2022):


(18) $$SD=\sqrt{\frac{1}{N-1}\overset{N}{\underset{n=1}{\sum }}{\left(x_{n}-mean\right)}^{2}}$$

#### Frequency Domain Analysis

Fourier Transform:


(19) $$F\left(\omega \right)=\overset{+\infty }{\underset{-\infty }{\int }}f\left(t\right)e^{-j\omega t}dt$$

Inverse Fourier transform:


(20) $$f\left(t\right)=\frac{1}{2\pi }\overset{+\infty }{\underset{-\infty }{\int }}F\left(\omega \right)e^{j\omega t}d\omega$$

#### Time-Frequency Domain Analysis

Satisfy:


(21) $$C_{\psi }=\int_{R}\frac{{\left|\hat{\psi }\left(\bar{\omega }\right)\right|}^{2}}{\left|\omega \right|}d\omega <\infty$$

Get a wavelet sequence:


(22) $$\psi_{a,b}\left(t\right)=\frac{1}{\sqrt{\left|a\;\right|}}\psi \left(\frac{t-b}{a}\right)\;\;a,b\in R;a\neq 0$$

## Analysis of Educational Mental Health and Emotional Perception

### Educational Psychology and Emotion Recognition

For colleges and universities, it is of positive significance to let students receive education, ensure their mental health, and manage and analyze their emotional state. Colleges and universities are places to deliver talents to the country and society. Colleges and universities should make students have no psychological burden, try to stimulate all their potential and realize their self-worth, so as to grow into useful people for society. Up to now, there are many theoretical researches on students’ mental health and emotional state in education. But these are relatively basic theoretical data. This paper is put forward under this premise. Aiming at students’ psychological and emotional recognition, we preliminarily constructed a recognition framework. Because of the diversity of human psychological emotions, we have designed a variety of different patterns for emotions, and we need to identify the patterns of emotions first. Then, a series of recognition processing are carried out, and finally the emotion analysis function is added instead of a single recognition function.

### Design Loss Function

Data are usually in continuous dimensions, and there is an imbalance. In order to solve this problem, we can use the loss function to reduce its influence. The smaller the value of loss function, the better the robustness of the model.

Calculate the combined loss of the two tasks. Then the total loss:


(23) $$L_{total}=L_{disc}+L_{cont}$$

(24) $$L_{disc}=\frac{1}{M\times C}\overset{M}{\underset{i=1}{\sum }}\overset{C}{\underset{j=1}{\sum }}\omega_{j}^{c}\left[\alpha_{j}p_{ij}\log{\overset{}{\hat{p}}}_{ij}+\beta_{j}\left(1-p_{ij}\right)\log\left(1-{\overset{}{\hat{p}}}_{ij}\right)\right]$$

(25) $$L_{cont}=\overset{M}{\underset{i=1}{\sum }}\overset{3}{\underset{k=1}{\sum }}\omega_{ik}^{cont}\left[{\left(\overset{}{\hat{y}}-y\right)}^{2}+\mathrm{||}\overset{}{\hat{y}}-y\mathrm{||}\right]$$

where *L_disc_* represents the loss function of discrete dimensions; *L_cont_* represents the loss function of continuous dimensions.

### Model Prototype Design

Convolution layer undertakes and produces a large number of computational parts in the network. Although the fully connected layer is retained, CNN can solve the obvious shortcomings of fully connected neural network. Enlightened by human visual nerve, CNN is more suitable for image processing. In addition to the function of preserving the effective features of images, it can also reduce the image with large data volume to small data volume.

Using cyclic neural network to solve the problem of data sequence. For the hidden layer, there are two parts to determining its value. One is the input layer at every moment; the other is the hidden layer of the previous moment. The specific formula is as follows:


(26) $$O_{t}=g\left(V\cdot S_{t}\right)$$

(27) $$S_{t}=f\left(U\cdot X_{t}+W\cdot S_{t-1}\right)$$

where *O_t_* represents the output at time *t*; *S_t_* represents the value of the hidden layer at time *t*. *W* is used to represent the weight matrix between each time point, and *W* is the same at each time in the whole training. In addition, for *S_t_*, its value depends not only on *X_t_*, but also on *S_t-1_*.

In this paper, a feature fusion model based on CNNID and LSTM is designed. Different data and network structures are input respectively, and then different fusion models are learned by combination. LSTM belongs to a kind of RNN, which is a better and more advanced variant RNN. With all the characteristics of RNN, the problems of gradient disappearance and explosion are improved, and the final model performance is better.

Therefore, this paper chooses the LSTM, which is better than the common version of RNN when designing the model. The two features are fused and spliced, and the calculation of full connection is carried out in order to obtain the classification prediction results. And when processing data, it is assisted by ant colony algorithm in computational intelligence to optimize the calculation of internal mechanism, improve the operation efficiency of the algorithm, and promote the search to be more efficient and effective.

## Experimental Analysis

### Build a Dataset

Collect the data set of other references, in order to ensure that the data collection is more in line with the research topic of this paper. Collect physiological data and facial data of these volunteers. These participants come from different majors from the same university, including undergraduate and master students. Because we tested mainly physiological signals, these 200 volunteers were ordinary people with good health, no physiological defects and good physical quality. Summary of related experimental equipment data.

When constructing data collection experiments, stress tests are needed for each person’s subjective scores. Assuming a stress level of 0 to 4, the volunteers were tested for how well they were awakened to the subjective stress of the experiment under different stimuli.

We chose to recruit 200 volunteers who volunteered to participate in this experiment. Among them, the proportion of men and women is 100 men and 100 women. Their average age is around 20 years old. If volunteers are under great pressure, they will have different feelings under different stimuli. This has a great influence on the accuracy of the data set finally collected and established in the experiment. Through the subjective scoring test, we can decide whether the current data are valid or not.

**Figure 1:**
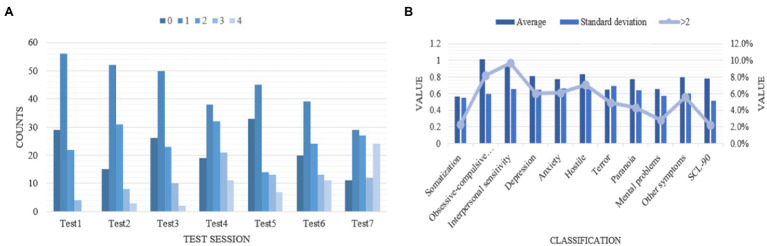
Performance and scores under mental health. **(A)** Subjective scores. **(B)** Mental health.

According to Figure 1A, we found that the first three tests have a very slight stress effect on the tester, which can be basically ignored. However, in the fourth, sixth and seventh tests, the stress increased significantly, and the subjective stress scores given by volunteers increased significantly. The final result shows that our data set is set up reasonably and can adopt these data.

According to the model, the students in a certain university are selected to test their mental health. The test results of this section show in Figure 1B. We can find that the average number of mental health indicators of students in this university is less than 2. This proves that all factors are negative and there is no positive. The overall students’ mental health is good, their emotions are stable and their stress is less. Among them, the average number of obsessive–compulsive disorder and interpersonal sensitivity is higher than 0.9, which is on the high side. Schools should pay more attention to these two aspects and strengthen related psychological prevention and enlightenment.

### Model Comparison Test

**Figure 2:**
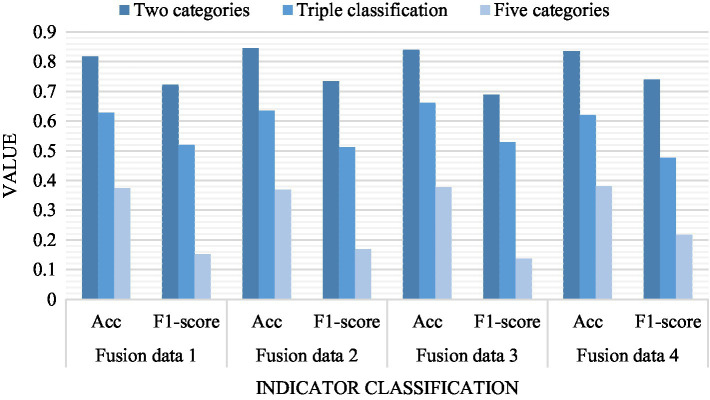
Results of different fusion data.

The model is used to test the accuracy (*Acc*, *F*1-score) of different fusion data. According to the results, the *Acc* index of data 2 is fused, and the accuracy rate of 2 classifications is as high as 0.8452. It is better than any other indicator of the rest of the data. Among the three categories, the *Acc* index of fusion data 3 has the highest accuracy, and the accuracy rate is as high as 0.6621. In the five categories, the highest accuracy rate is 0.3814, which is the *Acc* index in the fused data 4. Generally speaking, the accuracy of *Acc* index is generally higher than *F*1-score index. On the whole, each index of feature data fusion is better than the original single channel model, and the fusion data accuracy is higher. As shown in Figure 2.

### Emotion Analysis Test

**Figure 3:**
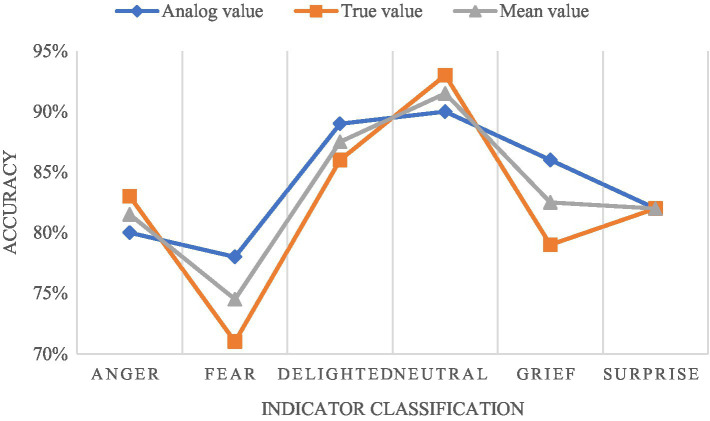
Comparison results of emotion test.

Make full use of the loss function value to test. By identifying the difference between participants’ psychological and emotional conditions and the real value, we can judge whether the value of the model results is infinitely close to the real situation. As long as the difference between the results and the real value is smaller, the better the model we designed is and the more accurate the emotional analysis is. In this section, we selected 100 emotional data for simulation test. Among them, there are six different emotions. We can find that surprise emotion is the most accurate, and the measured values are all 82%. The test values for sadness and fear are the biggest difference, and the accuracy difference is about 7%. The difference between the test results of other emotions is no more than 3%. As shown in Figure 3.

## Conclusion

This paper applies computational intelligence to educational psychology. The recognition model is established by physiological signals. The specific structure and recognition framework of the model are determined. And the model is applied to the specific detection. Use one or a group of data to describe the emotional characteristics of college students, and analyze and mine their psychological state. The article carries out model and analysis test. The purpose is to verify the application value of this emotional psychoanalysis model. The research results show that the final model of the experiment performs well and the research is successful, which has certain experimental reference value. The model in this paper is proposed based on deep learning, and the optimized computational intelligence changes the application effect of the model. However, there are still some problems and deficiencies in the research. We further look forward to the future work, so that the latecomers can learn and expand their applications. For example, the data set established in this paper is monotonous, and the quality and quantity of data need to be improved; The simulation times of the model are less, so it is necessary to increase the number of samples for training and reduce the degree of over-fitting; The model structure should also incorporate some better changes; For the study of emotion, we should strengthen human-computer interaction and provide more emotions and psychological states for machine learning. Effective feedback can help machines calibrate the accuracy of emotions more accurately; the research should not only be limited to physiological signals, but also expand the scope of data features. It is also an effective method to introduce emotion recognition research in voice, face, and limb movements.

## Data Availability Statement

The original contributions presented in the study are included in the article/supplementary material; further inquiries can be directed to the corresponding author.

## Ethics Statement

Ethical review and approval was not required for the study on human participants in accordance with the local legislation and institutional requirements. Written informed consent from the patients/participants or patients/participants legal guardian/next of kin was not required to participate in this study in accordance with the national legislation and the institutional requirements.

## Author Contributions

JL is mainly responsible for the conception, writing, and related analysis of the full text. HW is responsible for writing the article, providing some solutions and related analysis. All authors contributed to the article and approved the submitted version.

## Funding

This work was supported by Major Theoretical and Practical Issues in Philosophy and Social Sciences of Shaanxi Province research Project: Youth Ideal Personality View in the New Era (No. 2021ND0187); 2020 Project of the 13th Five-Year Plan of Education Science of Shaanxi Province: research on the positive emotion intervention strategies to improve the mental health of Rural Adolescents in Shaanxi Province (No.: SGH20Y1098); Xi’an 2021 Social Science Planning Fund Project (No.: FS58): The application research of inherits and promotes The Yan’an Spirit to psychological education in colleges and universities from the perspective of narrative.

## Conflict of Interest

The authors declare that the research was conducted in the absence of any commercial or financial relationships that could be construed as a potential conflict of interest.

## Publisher’s Note

All claims expressed in this article are solely those of the authors and do not necessarily represent those of their affiliated organizations, or those of the publisher, the editors and the reviewers. Any product that may be evaluated in this article, or claim that may be made by its manufacturer, is not guaranteed or endorsed by the publisher.
